# Bacterial diversity and community structure in the rhizosphere of four *Ferula* species

**DOI:** 10.1038/s41598-018-22802-y

**Published:** 2018-03-28

**Authors:** Xiuling Wang, Zhongke Wang, Ping Jiang, Yaling He, Yudi Mu, Xinhua Lv, Li Zhuang

**Affiliations:** 1 College of Life Sciences, Key Laboratory of Xinjiang Phytomedicine Resource Utilization, Ministry of Education, Shihezi University, Xinjiang Shihezi, 832003 China; 2 Agricultural college of, Shihezi University, Xinjiang Shihezi, 832003 China; 3 School of medicine, Shihezi University, Xinjiang Shihezi, 832003 China; 4 Faculty of Economics and Management, East China Normal University, Shanghai, 200062 China

## Abstract

The medicinal value of the *Ferula L*. has been recognized for more than a thousand years. Wild stocks of *Ferula* have declined dramatically because high economic value has led to overharvesting. The objective of this study was to compare the rhizosphere microbial community of four *Ferula* species [*F. syreitschikowii* K.-Pol., *F. gracilis* (Ledeb.) Ledeb., *F. ferulaeoides* (Steud.) Korov., and *F. lehmannii* Boiss.] in the northern part of Xinjiang, China. The 16S rRNA sequences of rhizosphere bacteria were obtained with an Illumina paired-end sequence platform. Analysis was conducted to determine the richness and diversity of the rhizosphere bacterial communities. Two-way ANOVA indicated that plant species and soil depth had no significant effect on the alpha diversity of rhizobacteria. Linear discriminant analysis effect size showed that *F. lehmannii* followed by *F. ferulaeoides* had the most biomarkers and the highest taxon level, *F. syreitschikowii* and *F. gracilis* the least, while *F. syreitschikowii* and *F. gracilis* had the least property. This trend is consistent with reports that the medicinal value of *F. lehmannii* and *F. ferulaeoides* is greater than that of *F. gracilis and F. syreitschikowii*. The results of this study provide information that could be used for the commercial cultivation of *Ferula* spp.

## Introduction

Twenty-six *Ferula* species and one *Ferula* variant are present in China^1^. Twenty of these species are found in the Xinjiang Uygur Autonomous Region, the center of origin of *Ferula L*. The medicinal value of *Ferula* L. was first recorded in *Materia Medica*, which was written in 659 A.D. during the Tang Dynasty. At least half of the *Ferula* species in China have high medicinal value, including *F. lehmannii* Boiss., *F. ferulaeoides* (Steud.) Korov., *F. fukanensis* K. M. Shen, and *F. sinkiangensis* K. M. Shen. *Ferula* species have been shown to have anti-cancer activity^2,3^, anti-inflammatory activity^4^, anti-bacterial activity^5,6^, anti-oxidant activity^7^ and anti-influenza activity^8,9^.

Because of their medicinal and economic value, wild *Ferula* has been over-harvested to the extent that *F. sinkiangensis* and *F. fukanensis* are now endangered. The technical of cultivating *Ferula spp*. have not been overcome, primarily because relatively little is known about the ecological characteristics of *Ferula L*.

Soil microorganisms, especially those in the rhizosphere, are an important ecological characteristic affecting plant growth and development^10^. The structure of rhizosphere microbial community is closely related to biological and abiotic factors^11–13^. These factors include soil type, plant species, and root zone location^14,15^. However, Singh *et al*. suggested that the effect of plant species on rhizosphere microbial community structure was relatively weak^16^. Soil fertility and tillage method are also very important factors affecting plant-microorganism interactions in cultivated systems^17,18^.

Numerous studies have shown that plant species specifically select microbial communities in the rhizosphere through rhizodeposition^16,19^. Growing roots provide root biomass and rhizodeposition for microorganisms. Therefore, the microbiota is generally different in rhizosphere soil than in bulk soil^20,21^. Microorganisms in the rhizosphere can be endophytic, epiphytic, or closely-associated. Furthermore, these microorganisms can have beneficial, neutral, or detrimental effects on plant growth^22^. Microbial populations can be many times greater in the rhizosphere than in the bulk soil and rhizosphere microorganisms have greater effect than bulk soil microorganisms on plant growth^23^.

The objective of this study was to compare the rhizosphere bacterial community of four *Ferula* species (*F. syreitschikowii* K.-Pol., *F. gracilis* (Ledeb.) Ledeb., *F. ferulaeoides*, and *F. lehmannii*) in the northern part of the Xinjiang Uygur Autonomous Region. Because they grow in arid environments, *Ferula* species have deep root systems. Therefore we wanted to know the effect of both species type and root depth on the rhizosphere microbial community. The results will increase understanding about the ecological characteristics of these four *Ferula* species, thus providing important information for their commercial production. We believe that this is the first study to use high throughput sequencing methods to analyze the rhizosphere microbial communities of *Ferula spp*. Previous studies used plate count methods^24,25^.

## Materials and Methods

### Site description and experimental design

Rhizosphere soil samples were collected from *F. syreitschikowii* (HDAW), *F. gracilis* (XJAW), *F. ferulaeoides* (DSAW), and *F. lehmannii* (DGAW) growing in separate fields between the end of May and the end of June in 2016. Table 1 describes the four sampling sites, all of which are in the northern Xinjiang Region.

**Table 1:** Description of the four sampling locations in the northern part of the Xinjiang Uygur Autonomous Region, China.

Location	Plant Species	Altitude	Latitude	Longitude
Hutubi County	*Ferula syreitschikowii* K.-Pol.	753 m	N44°00′19.2″	E86°51′47.0″
Fukang City	*Ferula gracilis* (Ledeb.) Ledeb.	541 m	N44°16′19.7″	E88°26′37.8″
Fuyun County	*F. ferulaeoides* (Steud.) Korov.	797 m	N46°35′54.7″	E88°35′53.1″
Shihezi City	*Ferula lehmannii* Boiss.	1030 m	N43°57′58.2″	E85°54′45.3″

The rhizosphere soil was collected using Riley and Barber’s shake method^26,27^: First, the entire root system of three *Ferula* plants was excavated from the soil profile. The plants were gently shaken to remove the bulk soil, and then rhizosphere soil samples were collected from approximately the 3, 20, and 40 cm root depths. The samples were labeled using a two-number system, where the first number indicates the depth (1, 2, and 3 represent the 3, 20, and 40 cm depths respectively) and the second number represents the replicate number. For example, DGAW2.3 represents the third replicate from 20 cm depth of the *F. syreitschikowii* root system. A total of 36 rhizosphere soil samples were analyzed (4 sites × 3 plants per site × 3 soil depths). The replicates were averaged to form 12 groups (4 sites × 3 soil depths) for some analyses. The rhizosphere soil samples were preserved using liquid N and sent to the Beijing Compass Biotechnology Co., Ltd. to perform high throughput sequencing.

### DNA extraction, amplification, and sequencing of 16S r RNA genes

Total genomic DNA was extracted from the samples using a centrifugal-type soil genomic DNA extraction kit. The concentration and purity of the DNA was monitored on 1% agarose gels. The DNA was diluted to a concentration of 1 ng μL^−1^ using sterile water. The 16S rRNA genes of the V4 region were amplified using 515F-806R (5′-GTGCCAGCMGCCGCGGTAA-3′ and 5′-GGACTA CHVGGGTWTCTAAT-3′) with barcodes. All PCR reactions were carried out with Phusion^®^ High-Fidelity PCR Master Mix (New England Biolabs).

The X1 loading buffer containing SYB green was mixed in equal volumes with the PCR products and then electrophoresed on 2% agarose gel for detection. Samples with a bright main strip between 400 and 450 bp were chosen for further experiments. The PCR products were mixed in equidensity ratios. The mixed PCR products were then purified with a Qiagen Gel Extraction Kit (Qiagen, Germany). Sequencing libraries were generated using a TruSeq^®^ DNA PCR-Free Sample Preparation Kit (Illumina, USA) following the manufacturer’s recommendations and index codes were added. The library quality was assessed on a Qubit 2.0 Fluorometer (Thermo Scientific) and an Agilent Bioanalyzer 2100 system. Finally, the library was sequenced on an Illumina HiSeq. 2500 platform which generated 250 bp paired-end reads.

Paired-end reads were assigned to samples based on their unique barcode and truncated by cutting off the barcode and primer sequence. Paired-end reads were merged using FLASH (V1.2.7)^28^, a very fast and accurate analysis tool designed to merge paired-end reads when at least some of the reads overlap the read generated from the opposite end of the same DNA fragment. The splicing sequences were called raw tags. Quality filtering of the raw tags was performed under specific filtering conditions to obtain high-quality clean tags according to the QIIME (V1.7.0)^29^ quality control process^30^. The tags were compared with Genomes OnLine Database (GOLD). The UCHIME algorithm was used to detect chimera sequences, and then the chimera sequences were removed to obtain the Effective Tags^31^.

Uparse software (Uparse v7.0.1001)^32^ was used for sequence analysis. The OTUs with > 97% similarity were screened for further annotation. The taxonomic information for each representative sequence was annotated using the Green Gene Database^33^ based on the RDP classifier (Version 2.2) algorithm^34^. Multiple sequence alignment was conducted using MUSCLE software (Version 3.8.31) to study the phylogenetic relationship of different OTUs and the differences among the dominant species among the 36 samples^35^.

### Soil Physicochemical Properties

The following soil properties were determined according to methods described by Bao *et al*.^36^, gravimetric soil water content, organic matter content (KCr_2_O_7_ method), total N (HClO_4_-H_2_SO_4_ digestion method), total P (Mo-Sb colorimetric method), total K (atomic absorption spectrometry), available N (CaCl_2_ extracts analyzed with a flow analyzer), available P (NaHCO_3_ extracts analyzed with the Mo-Sb colorimetric method), available K (NH_4_OAc extracts analyzed by atomic absorption spectrometry), pH (1:5 soil:water), electric conductivity (1:5 soil:water) and total dissolvable salts (atomic absorption spectrometry and titration methods).

### Statistical analyses

The OTU-abundance information was normalized using the sequence number corresponding to the sample with the fewest sequences (i.e., XJAW2.2). The alpha diversity and beta diversity were subsequently performed using the normalized data.

Alpha diversity was applied to analyze species diversity in a sample through six indices: observed-species, Chao1, Shannon, Simpson, ACE, and Good’s coverage. All of these indices were calculated with QIIME (Version 1.7.0). Community richness was identified using the Chao1, ACE, Shannon and Simpson estimators. Community diversity was identified using the Shannon and Simpson indexes. Sequencing depth was characterized by Good’s coverage.

Beta diversity analysis was used to evaluate differences in species complexity among the samples. Beta diversities based on both weighted and unweighted Unifrac were calculated by QIIME software (Version 1.7.0). Unweighted Pair-group Method with Arithmetic Means (UPGMA) clustering was performed as a type of hierarchical clustering method to interpret the distance matrix using average linkage. The UPGMA clustering was conducted by QIIME software (Version 1.7.0). Linear discriminant analysis (LDA) effect size (LEfSe) was calculated online, the web site for online analysis is: https://huttenhower.sph.harvard.edu/galaxy/.

Statistical analysis was carried out with SPSS 19.0 (IBM Inc., Armonk, USA). Two-way ANOVA was used to analyze the effects of different soil depths and *Ferula* species on the soil physicochemical properties and bacterial abundance data. Pearson correlations (r) were also run among the soil physicochemical factors and bacterial abundance data. The data sets generated during this study are publicly available. More data can be obtained from the corresponding author.

## Results

### Sequencing Results and Quality Control

**Figure 1:**
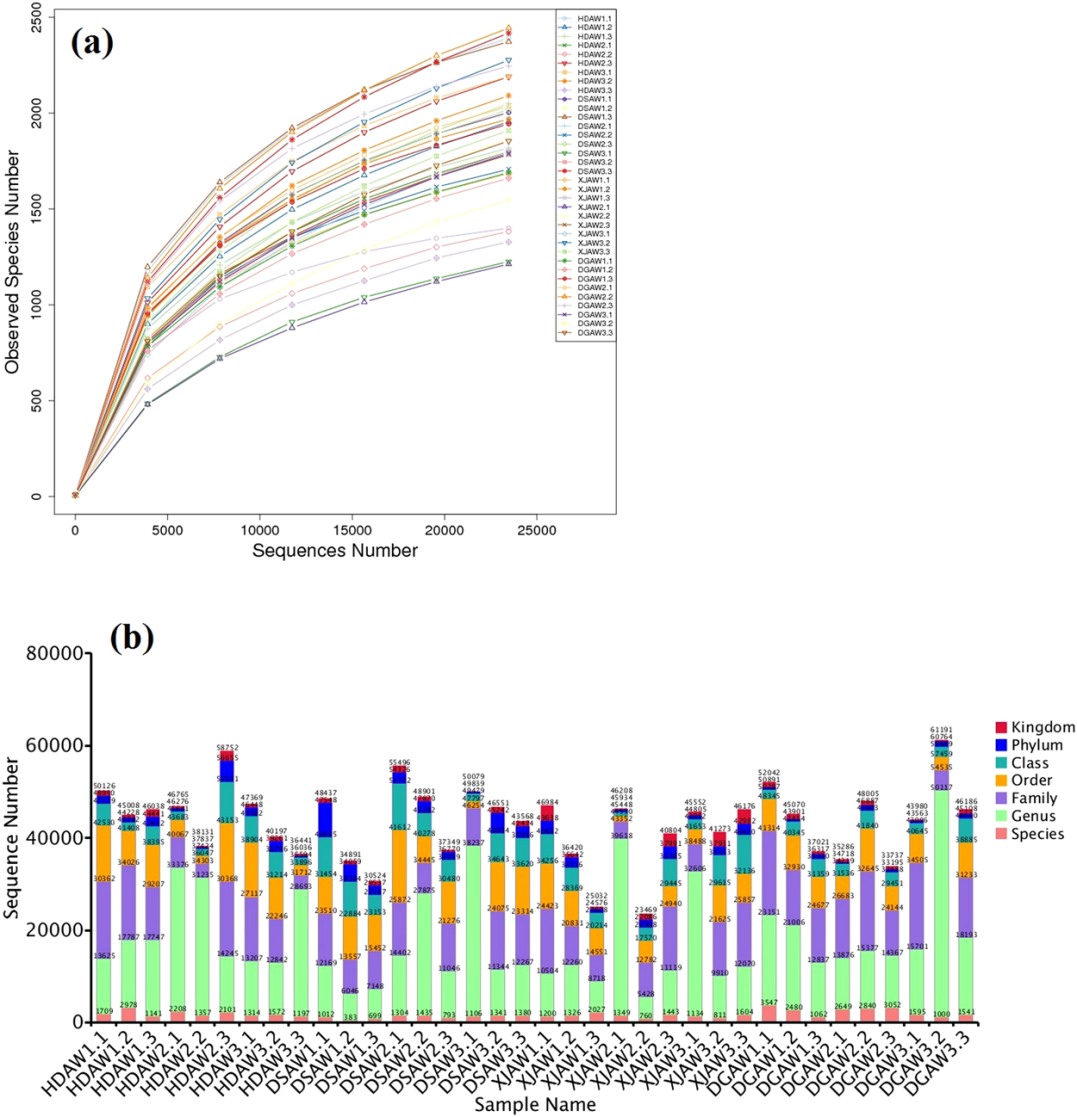
(**a**) Rarefaction curves of OTUs at 97% similarity for each sample and (**b**) Distribution of the number of tags on each classification level (k, p, c, o, f, g, s). Abbreviations: HDAW, *F. syreitschikowii*; XJAW, *F. gracilis*; DSAW, *F. ferulaeoides;* and DGAW, *F. lehmannii*. The first number indicates the root depth (1, 2, and 3 represent the 3, 20, and 40 cm depths, respectively) and the second number represents the replicate number.

After filtering out low-quality and short sequence reads, a total of 1,559,059 raw sequences were obtained from 36 soil samples. Rhizosphere soil from the 40-cm root depth of *F. lehmannii* had the greatest abundance (Table S2, 50,452 sequences, average of three replications). Rhizosphere soil from the 3-cm root depth of *F. gracilis* had the lowest abundance (36,145 sequences, average of three replications). The rarefaction curves of all 36 samples were generated by QIIME software (Version 1.7.0) and normalized to the minimum number of sequences (23,469 sequences in XJAW2.2) (Fig. 1a).

The distribution of bacteria in each taxonomic category is shown in Fig. 1b. In the rhizosphere soil of the four *Ferula* species, 97.4% of the sequences were assigned to the phylum and class level, 82.7% to the order level, 66.0% to the family level, 40.9% to the genus level, and 3.7% to the species level by the Illumina HiSeq. 2500 platform.

### Soil Bacterial Communities

**Figure 2:**
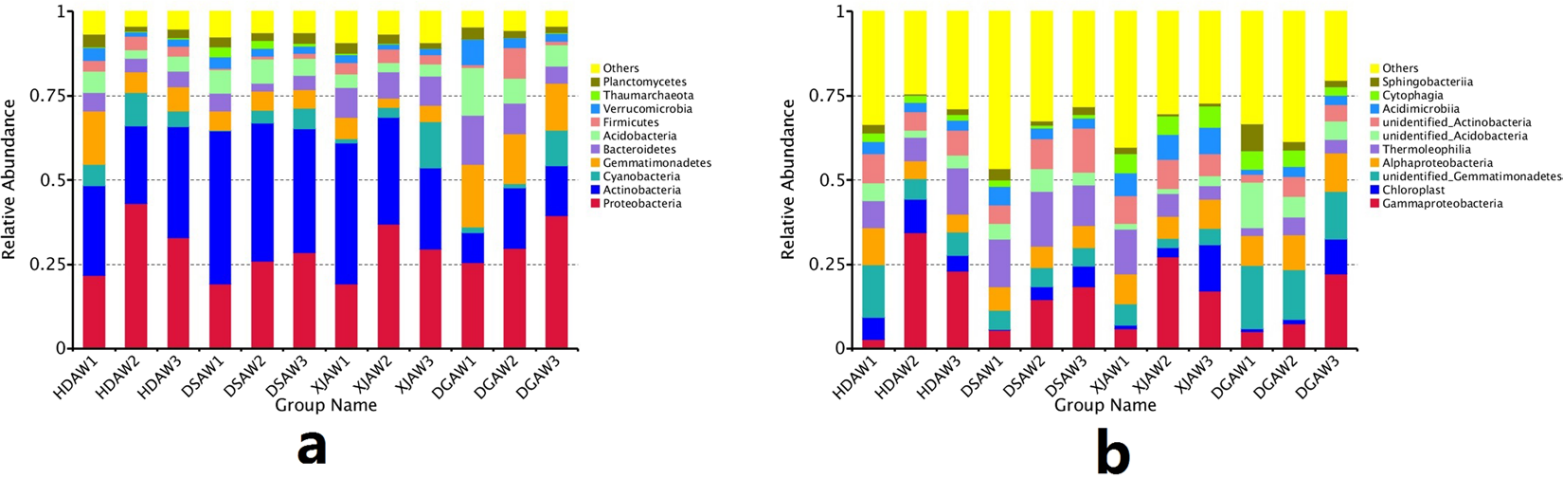
The relative abundance of the top ten phyla (**a**) and genera (**b**) in the *Ferula* rhizosphere. Abbreviations: HDAW, *F. syreitschikowii*; XJAW, *F. gracilis*; DSAW, *F. ferulaeoides;* and DGAW, *F. lehmannii*. The numbers indicate the root depth (1, 2, and 3 represent the 3, 20, and 40 cm depths, respectively).

Figure 2a shows the top ten bacterial phyla in the rhizosphere soil. Proteobacteria and Actinobacteria had the greatest relative abundance among the top ten phyla in this study (Fig. 2a). Summed together, Proteobacteria and Actinobacteria accounted for more than 50% of the relative abundance in major simples (27). Previous studies have shown that Proteobacteria and Actinobacteria have the greatest abundance in many soil microbial communities including grassland, mangrove wetland, and cucumber fields^16,37,38^. Two-way ANOVA indicated that *Ferula* species significantly affected the relative abundance of Actinobacteria (*P* < 0.01), Gemmatimonadetes (*P* < 0.01), Bacteroidetes (*P* < 0.01), Acidobacteria (*P* < 0.01), Verrucomicrobia (*P* < 0.05) and Thaumarchaeota (*P* < 0.01). Soil depth significantly affected the relative abundance of Proteobacteria (*P* < 0.05), Acidobacteria (*P* < 0.05), Firmicutes (*P* < 0.05) and Verrucomicrobia (*P* < 0.01).

Figure 2b shows the top ten bacterial classes in the rhizosphere soil. Gammaproteobacteria had the highest abundance (15.28%), followed by unidentified_Gemmatimonadetes (8.90%) and Thermoleophilia (8.89%). The relative abundance of unidentified_Gemmatimonadetes (*P* < 0.01), Thermoleophilia(*P* < 0.05), unidentified_Acidobacteria (*P* < 0.01), unidentified_Actinobacteria (*P* < 0.05), Acidimicrobiia (*P* < 0.01), Cytophagia (*P* < 0.05) and Sphingobacteriia (*P* < 0.01) were significantly affected by Ferula species. The abundance of Sphingobacteriia was significantly affected by soil depth (*P* < 0.01).

### Soil Physicochemical Properties

Soil physicochemical properties vary among soil types and with soil depth. Therefore, we measured 12 common soil physiochemical properties (Table S1) and then analyzed the correlation between these properties and the relative abundance of bacteria at the phylum and class levels. Two-way ANOVA indicated that Ferula species and soil depth significantly affected all 12 Soil physicochemical properties (*P* < 0.01).

By factor analysis, we reduced 12 physicochemical factors to 4 principal components. The results of the correlation analysis of these four components with the relative abundance of bacteria in the rhizosphere are shown in Table S2 (phylum level) and Table S3 (class levels). We found that 12 physicochemical factors in rhizobacteria also have an impact on each other. For example, nitrate nitrogen and ammonium nitrogen are closely related, and they have impact on certain bacteria consistently.

Overall, these results suggest that soil properties were positively correlated with the relative abundance of bacteria.

### Alpha Diversity

Alpha Diversity is used to analyze the diversity of species within community samples^39^. In general, sequences with greater than 97% identity are clustered into one operational taxonomic unit. The Alpha Diversity analysis index for different samples at a 97% identity are shown in Table 2. Two-way ANOVA indicated no significant differences among soil samples in observed species, Shannon, Simpson, Chao1, and ACE. Good’s coverage ranged from 97 to 98%, indicating that the measurement depth has met the requirements.

**Table 2:** Estimated OTU richness and diversity indices of the rhizosphere soil collected from *Ferula*. L in the northern part of Xinjiang Uygur Autonomous Region of China.

**Sample name**	**Observed species**	**Shannon**	**Simpson**	**Chao1**	**ACE**	**Good’s coverage**
HDAW1	2132.67	8.82	0.99	2807.06	2876.83	0.97
HDAW2	1790.67	6.98	0.91	2419.85	2413.69	0.97
HDAW3	1871.00	7.82	0.94	2438.01	2481.69	0.97
XJAW1	1684.67	8.47	0.99	2059.66	2088.97	0.98
XJAW2	1621.33	7.07	0.91	3143.05	2470.74	0.97
XJAW3	1994.67	7.78	0.95	2767.36	2866.19	0.97
DSAW1	2061.33	9.03	0.99	2449.86	2537.20	0.98
DSAW2	1848.67	8.32	0.98	2375.49	2404.49	0.98
DSAW3	1814.33	7.76	0.97	2463.51	2534.72	0.97
DGAW1	1765.00	8.44	0.99	2314.67	2361.15	0.98
DGAW2	2242.00	9.24	0.99	2789.28	2827.43	0.97
DGAW3	1730.67	7.04	0.93	2507.36	2557.33	0.97

Abbreviations: HDAW, *F. syreitschikowii*; XJAW, *F. gracilis*; DSAW, *F. ferulaeoides;* and DGAW, *F. lehmannii*. The numbers indicate the root depth (1, 2, and 3 represent the 3, 20, and 40 cm depths, respectively).

### Petal Map Based Operational Taxonomic Units

**Figure 3:**
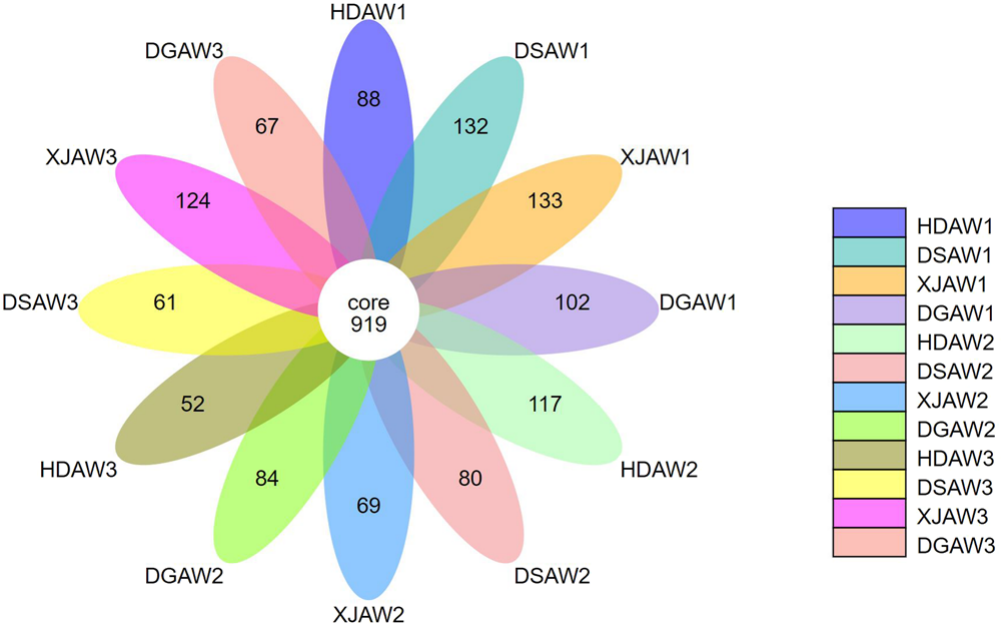
Petal Map Based Operational Taxonomic Units. Each petal in the petal map represents one sample, with different colors representing different samples. The middle core number represents the common OTUs of all samples, and the number on the petals represents the Unique OTU number of each sample. Abbreviations: HDAW, *F. syreitschikowii*; XJAW, *F. gracilis*; DSAW, *F. ferulaeoides;* and DGAW, *F. lehmannii*. The numbers indicate the root depth (1, 2, and 3 represent the 3, 20, and 40 cm depths, respectively).

Each petal in the petal map represents one sample, with different colors representing different samples. The middle core number represents the common operational taxonomic units of all samples, and the number on the petals represents the Unique operational taxonomic units number of each sample. We can see 919 core bacteria perational taxonomic units in Fig. 3. We speculate that the 919 core bacteria are closely related to the growth of *Ferula L*. and may promote the growth of the wild *Pleurotus ferulae Lanzi*. Wild *Pleurotus ferulae Lanzi* is an edible fungus that is simply sent or saprophytic to the roots of *Ferula L*. In addition, these core bacteria may provide a reference to soil microorganisms in artificially cultivated Ferula. In addition, these core bacteria may provide a reference to soil microbiology for the artificial cultivation of *Ferula L*. plants.

### Beta Diversity

In the Beta diversity study, the Weighted Unifrac distance and the Unweighted Unifrac distance were used to measure the dissimilarity coefficient between two samples. The smaller the value, the smaller the differences in species diversity between the two samples.

**Figure 4:**
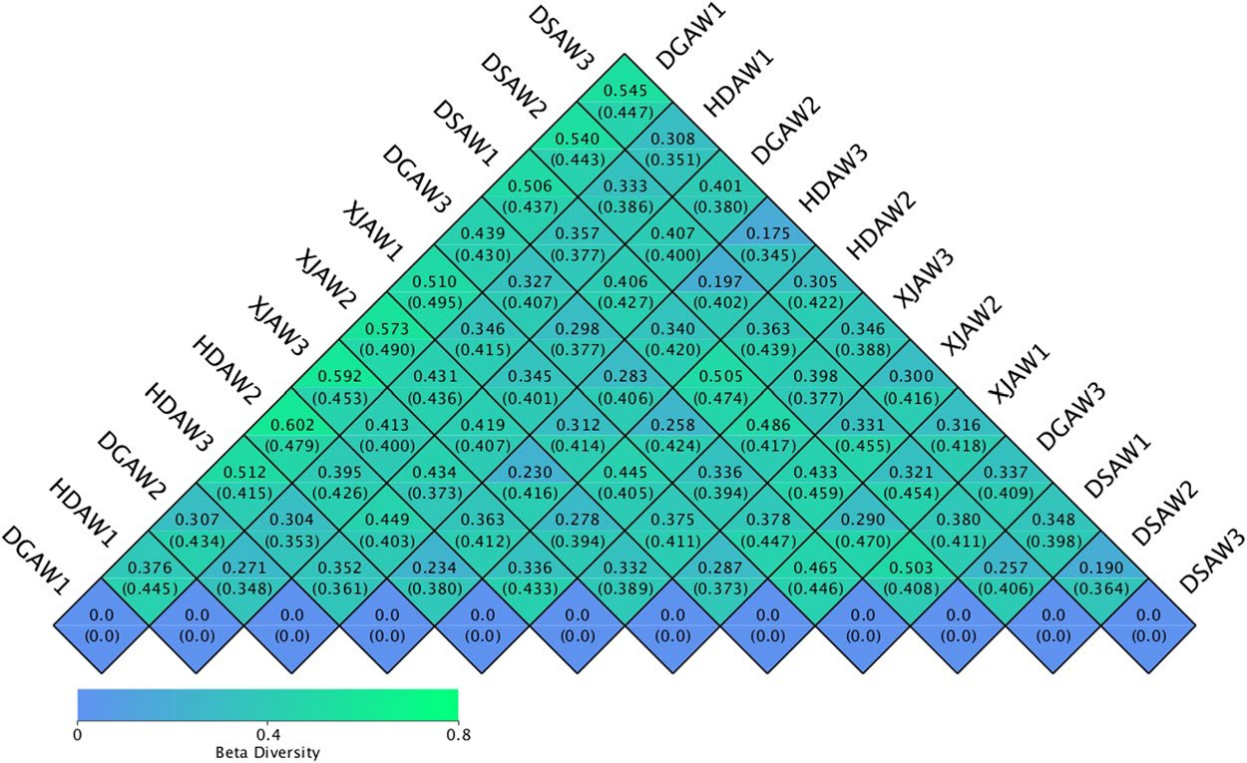
Beta diversity heat map based on the UniFrac distance. The upper triangle is the weighted distance. The distance from the lower triangles is the unweighted distance. Abbreviations: HDAW, *F. syreitschikowii*; XJAW, *F. gracilis*; DSAW, *F. ferulaeoides;* and DGAW, *F. lehmannii*. The numbers indicate the root depth (1, 2, and 3 represent the 3, 20, and 40 cm depths, respectively).

Figure 4 shows a heat map of the beta diversity measurements. In general, the distances of samples based on weighted-Unifrac were between 0.175 and 0.602. The distances of the samples based on unweighted-Unifrac were between 0.345 and 0.495. Overall, the data of Unifrac distances is relatively small, therefore, there are some differences in rhizosphere microbial communities between different species and different depths of soil, but this difference is not so obvious. The bacterial communities in the three replicates were highly similar, which is probably because the samples were collected from close proximity.

### Cluster analysis

**Figure 5:**
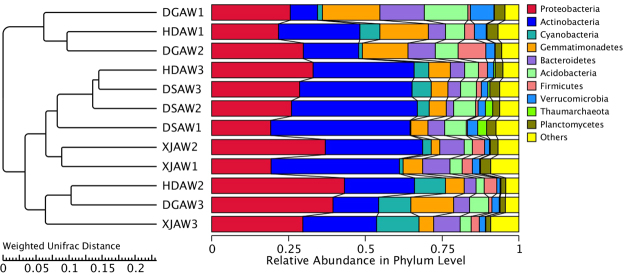
UPGMA tree based on Weighted UniFrac Distance at the phylum level. Explanation: On the left is the UPGMA cluster tree structure, on the right is the species relative abundance distribution at the phylum level for each sample. The phylogenetic relationship of the bacterial isolates was determined by PCR sequencing portions of the 16S rRNA gene. The phylogenetic tree showed that the bacterial communities were divided into three major groups, with each group containing samples from different soil depths and different *Ferula* species. The samples within each group were always from adjoining depths (i.e., either the 3 and 20 cm depths or the 20 and 40 cm depths). This indicated that soil depth had some effect on bacterial community structure. The bacterial communities in the three replicates were highly similar, which is probably because the samples were collected from close proximity. Abbreviations: HDAW, *F. syreitschikowii*; XJAW, *F. gracilis*; DSAW, *F. ferulaeoides;* and DGAW, *F. lehmannii*. The numbers indicate the root depth (1, 2, and 3 represent the 3, 20, and 40 cm depths, respectively).

In order to study the similarity between different samples, we performed a cluster analysis of the samples and constructed a sample cluster tree. In environmental biology, UPGMA (Unweighted Pair-group Method with Arithmetic Mean) is a commonly used clustering analysis method, which is used to solve the classification problem. The UPGMA cluster analysis was performed using the Weighted Unifrac distance matrix and the Unweighted Unifrac distance matrix, and the clustering results were integrated with species relative abundance column chart at phyla taxon level (Fig. 5) for each sample.

### Linear Discriminant Analysis Effect Size

**Figure 6:**
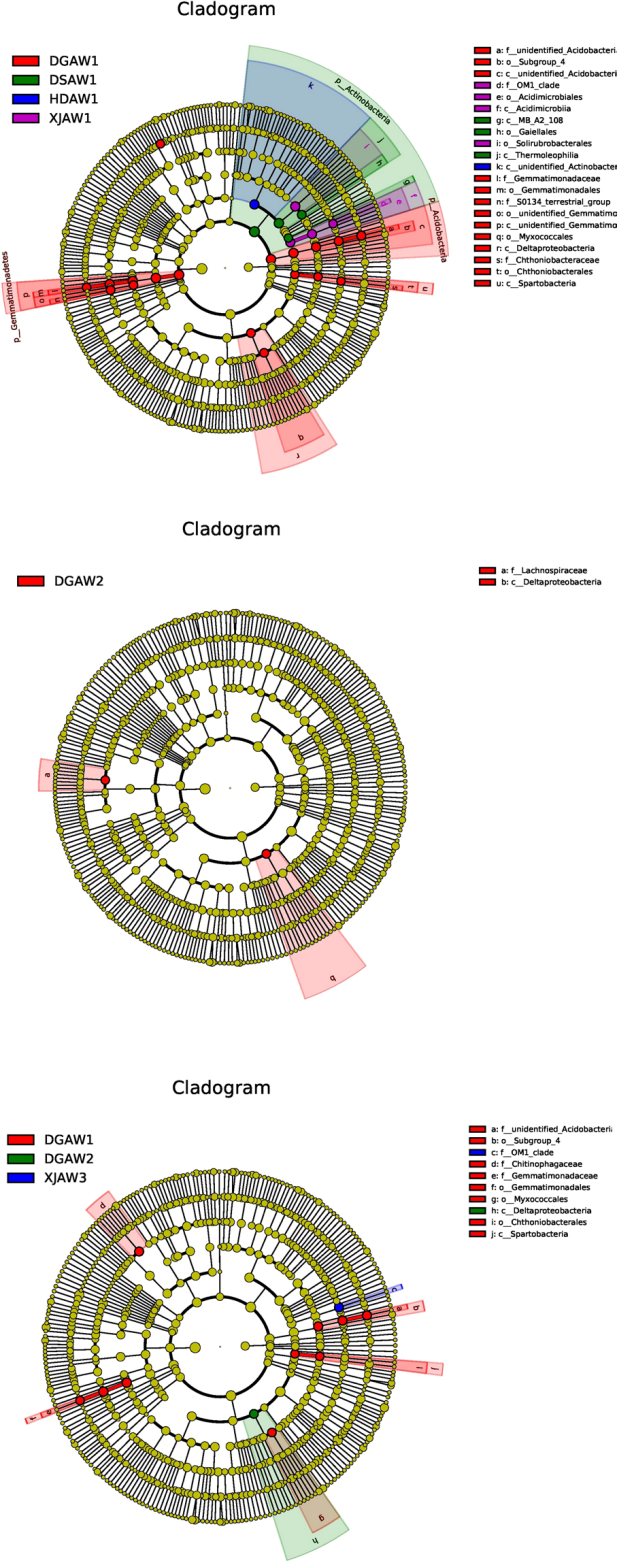
Cladograms indicating the polygenetic distribution of bacterial lineages in the rhizosphere of four Ferula species as determined by linear discriminant analysis (LDA) effect size (LEfSe). (**a**) The uppermost (3 cm) root depth; (**b**) the intermediate (20 cm) root depth; and (**c**) all root depths (3, 20, and 40 cm). Coloring principles: Species with no significant difference were uniformly colored yellow, the species of Biomarker were colored following the different group, the red node was the bacteria group that played an important role in the red group, and the green node was indicated to play an important role in the green group bacterial taxa. The name of the species represented by the English letters in the figure is shown in the illustration on the right. Abbreviations: HDAW, *F. syreitschikowii*; XJAW, *F. gracilis*; DSAW, *F. ferulaeoides;* and DGAW, *F. lehmannii*. The numbers indicate the root depth (1, 2, and 3 represent the 3, 20, and 40 cm depths, respectively). Indicators were defined as those with an LDA > 3. Red, green, blue, and purple circles represent bacterial biomarkers in *F. lehmannii, F. ferulaeoides*, *F. syreitschikowii*, and *F. gracilis*, respectively.

The LEfSe (LDA Effect Size) analysis was able to search for statistically significant Biomarker^40^ between groups, i.e. species with significant differences between groups. Lefse statistical results include three parts, namely, the LDA value distribution histogram, evolutionary branch graph (phylogenetic distribution) and biomarker abundance comparison chart in different groups. The article shows the evolution of the branch map only. We used LEfSe to identify discriminative taxon among different *Ferula* species and soil depths. First, the LEfSe analysis of the rhizophere bacteria was done for the four *Ferula* species at the same soil depth. An LDA score of 3 was used to identify bacterial groups with statistical significance. In the uppermost root depth (3 cm), the LDA scores of 27 taxa were greater than 3: *F. Lehmannii* (18 taxa), *F. ferulaeoides* (4 taxa), *F. gracilis* (4 taxa), and *F. syreitschikowii* (1 taxon) (Fig. 6a). At the intermediate root depth (20 cm), two taxa exhibited significant differences; both of them were in *F. lehmannii* (Fig. 6b). No discriminative taxa were observed in the lowest (40 cm) root depth.

The LEfSe of all species and depths showed 12 bacterial taxa with significant differences. Ten of the taxa were in the uppermost (3 cm) root depth of *F. lehmannii*, one taxon was in the intermediate (20 cm) root depth of *F. lehmannii*, and one taxon was in the lowest (40 cm) depth of *F. gracilis* (Fig. 6c). Overall, *F. lehmannii* had more biomarkers than the other *Ferula* species, especially in the uppermost (3 cm) root depth.

## Discussion

Many *Ferula* species have significant medicinal value, however they are difficult to cultivate. Information about the ecological characteristics of *Ferula* would be helpful for developing its commercial production. Rhizosphere microbial populations are an important factor influencing plant growth. Therefore, the objective of this study was to learn more about the ecological characteristics of four *Ferula* species by analyzing rhizosphere bacterial diversity and community structure at three root depths of four *Ferula* species. The result of cluster indicated that soil depth had some effect on bacterial community structure. The LEfSe (LDA Effect Size) analysis show that the number of biomarkers decreased significantly as soil depth increased. This show that some bacteria are more sensitive to soil depth, and the quantitative advantage of these bacteria becomes less pronounced as the depth of the soil increased. Linear discriminant analysis effect size showed that *F. lehmannii* followed by *F. ferulaeoides* had the most biomarkers and the highest taxon level, *F. syreitschikowii* and *F. gracilis* the least. This trend is consistent with reports that the medicinal value of F. lehmannii and F. ferulaeoides is greater than that of *F. gracilis* and *F. syreitschikowii*^1^. This shows that some rhizosphere bacteria are sensitive to soil depth and medicinal value of Ferula. Rhizosphere bacteria populations are affected by compounds and energy released by roots^20,41^. *Ferula* plants are rich in resin, volatile oil (thioether compounds), and many other physiologically active ingredients, including monoterpene coumarins, sesquiterpene coumarins, sesquiterpenes, furanocoumarins and aromatic compounds^9,42^.

In summary, we propose a hypothesis that the reason that some of the soil bacteria are sensitive to soil depth and medicinal value is that these microorganisms are sensitive to volatile oil from plant.

As the rhizosphere soil volatile substances can not only come from the roots, but also from the ground parts of Ferula. For example, shoots can emit volatile substances in gaseous form which can diffuse into the surface soil, the rainwater can dissolve the volatile material to bring it into the soil. These processes gradually become less pronounced with the soil depth increased. So the abundance of some volatile-sensitive bacteria is higher in the upper soil (3 cm) and lower in the deeper soil (20 and 40 cm).

The results of LEfSe suggest that *Ferula* species with greater medicinal efficacy have a more unique microbial community (biomarkers) in the rhizosphere. The likely reason is that *Ferula* species with high medicinal efficacy produce more and better *Ferula* gum and volatile substances than species with low medicinal efficacy. That is to say, the medicinal value of Ferula is closely related to this volatile substance. The more volatile material the flavor has, the higher medicinal value is. We hypothesize that the differences in microbial populations among *Ferula* species were primarily due to differences in the production of volatile substances which can increase microbial diversity and abundance.

One shortcoming in our study is that the four *Ferula* species in this study were collected from areas which differed in geographical environment and climatic conditions. Ecological and geographical conditions greatly influence the terpenoid content of *Ferula* plants^43^. Therefore, the plant species factor in this study also includes the effects of the different environments in which the plants were growing. However, it is rare to find two *Ferula* species in the same field. We are not aware of any places where *Ferula* is being cultivated, so we could not obtain *Ferula* plants that had been grown under identical conditions. Each *Ferula* species has its unique habitat. Thus to a certain extent, the four *Ferula* species represent the environment in which they were growing.

Previous studies have examined the rhizosphere microbial community of *F. sinkiangensis* and *F. fukanensis*^24,25^. This study was the first to describe the rhizosphere microbial community of *F. syreitschikowii*, *F. gracilis*, *F. ferulaeoides*, and *F. lehmannii*. Many studies have compared the rhizosphere microbial community among species within a genus. Liu proposed that tree species influenced microbial diversity and nitrogen availability in rhizosphere soil^44^. Ladygina and Hedlun observed that plant species influenced microbial diversity and carbon allocation in the rhizosphere soil^45^. Cleary reported that root depth and plant species influenced microbiological parameters and bacterial composition in a mercury contaminated salt marsh^46^.

In conclusion, the results indicate that soil depth had some effect on bacterial community structure. And some rhizosphere bacteria are sensitive to soil depth and medicinal value of Ferula. The abundance of specific rhizosphere bacteria become higher as the medicinal value of the *Ferula* species increased and the soil depth decreased. This discovery provides insight about the ecological characteristics of *Ferula*. More information is still needed, but perhaps we can increase the survival rate of cultivated *Ferula* by artificially increasing the abundance of certain microorganisms.

## Electronic supplementary material

supplementary information
