# Supplementary material for: Bacterial diversity and community structure in the rhizosphere of four Ferula species
Source: Sci Rep. 2018 Mar 28;8:5345. doi: 10.1038/s41598-018-22802-y (PMC5871818; doi:10.1038/s41598-018-22802-y)
Supplement: Supplementary file 1 — supplementary information [file 41598_2018_22802_MOESM1_ESM.pdf]

Bacterial diversity and community structure in the rhizosphere of four *Ferula* species.

Xiuling Wang[1], Zhongke Wang[1], Ping Jiang[2], Yaling He[3], Yudi Mu[4], Xinhua Lv[1], Li Zhuang*[1]

[1]College of Life Sciences, Key Laboratory of Xinjiang Phytomedicine Resource Utilization, Ministry of Education, Shihezi University, Xinjiang Shihezi 832003, China.

[2]Agricultural college of Shihezi University, Xinjiang Shihezi 832003, China.

[3]School of medicine, Shihezi University, Xinjiang Shihezi 832003, China.

[4]Faculty of Economics and Management, East China Normal University, Shanghai 200062, China

* Correspondence to: Li Zhuang (3462352867@qq.com)

Sample	**SM** (%)	**OM** (g/kg)	**TN** g/kg	**TP** g/kg	**TK** g/kg	**Nitrate-N** mg/kg	**Ammonium-N** mg/kg	**Olsen-P** mg/kg	**AK** mg/kg	**pH**	**EC** mS/cm	**TDS** g/kg
HDAW1	5.01	11.98	0.73	0.92	20.11	7.00	21.92	8.62	372.00	8.76	0.18	0.95
HDAW2	5.55	8.88	0.63	0.78	20.55	36.37	16.48	6.28	156.98	8.98	0.63	1.88
HDAW3	5.49	9.06	0.62	0.76	20.10	59.92	13.03	4.34	110.43	8.00	1.12	3.23
XJAW1	3.37	8.40	0.58	0.89	19.49	5.31	15.74	6.60	387.08	7.74	2.01	6.65
XJAW2	4.41	7.39	0.49	0.65	19.01	8.15	16.16	1.84	234.85	8.26	1.83	5.03
XJAW3	5.51	7.11	0.45	0.62	18.19	7.75	13.26	2.55	182.64	7.76	2.58	6.88
DSAW1	1.59	4.26	0.30	0.74	19.76	4.91	11.70	7.68	461.46	8.74	0.15	0.75
DSAW2	2.88	7.37	0.42	0.55	20.90	4.16	13.33	5.74	133.96	8.66	0.29	0.98
DSAW3	2.55	4.54	0.31	0.46	17.94	3.49	12.19	4.25	70.30	8.45	0.38	1.03
DGAW1	9.92	8.33	0.44	0.59	21.38	5.74	10.89	1.29	320.57	9.93	0.50	2.70
DGAW2	11.61	5.51	0.43	0.64	22.93	5.46	12.96	0.98	307.68	10.08	0.77	4.40
DGAW3	11.71	4.79	0.40	0.74	21.07	7.69	12.08	0.80	326.74	10.03	0.86	4.35

Table S1. Physicochemical properties of rhizosphere soil of four *Ferula* species. Abbreviations: HDAW, *F. syreitschikowii*; XJAW, *F. gracilis*; DSAW, *F. ferulaeoides*; and DGAW, *F. lehmannii*. The numbers 1, 2, and 3 indicate root depths of 3, 20, and 40 cm, respectively. SM, soil moisture; OM, organic matter; TN, total N; TP, total P; TK, total K; AK, available K, EC, electrical conductivity; TDS, total dissolved salts.

Sample Name	Kingdom	Phylum	Class	Order	Family	Genus	Species
HDAW1.1	50126	48990	47279	42530	30362	13625	1709
HDAW1.2	45008	44228	43282	41408	34026	17787	2978
HDAW1.3	46038	44441	42432	38395	29207	17747	1141
HDAW2.1	46765	46276	45641	43683	40067	33376	2208
HDAW2.2	38131	37837	37424	36047	34303	31235	1357
HDAW2.3	58752	56655	52031	43153	30368	14245	2101
HDAW3.1	47369	46448	44582	38904	27117	13207	1314
HDAW3.2	40197	39261	36796	31214	22246	12842	1572
HDAW3.3	36441	36036	35594	33896	31712	28693	1197
DSAW1.1	48437	47548	40025	31454	23510	12169	1012
DSAW1.2	34891	34059	30364	22884	13557	6046	383
DSAW1.3	30524	29537	27547	23153	15452	7148	699
DSAW2.1	55496	54126	51772	41612	25872	14402	1304
DSAW2.2	48901	47870	45292	40278	34445	27875	1435
DSAW2.3	37349	36770	35059	30480	21276	11046	793
DSAW3.1	50079	49839	49479	47797	46254	38237	1106
DSAW3.2	46551	45242	40954	34643	24075	11344	1341
DSAW3.3	43568	42474	39916	33620	23314	12267	1380
XJAW1.1	46984	43638	40652	34256	24423	10504	1200
XJAW1.2	36420	35642	33526	28369	20831	12260	1326
XJAW1.3	25032	24576	23628	20214	14551	8718	2027
XJAW2.1	46208	45934	45448	44530	43352	39618	1349
XJAW2.2	23469	22086	20368	17570	12782	5428	760
XJAW2.3	40804	37991	35305	29445	24940	11119	1443
XJAW3.1	45552	44805	43952	41653	38488	32606	1134
XJAW3.2	41273	37931	36173	29615	21625	9910	811
XJAW3.3	46176	42982	40620	32136	25857	12070	1604
DGAW1.1	52042	50891	50287	48345	41314	23151	3547
DGAW1.2	45070	43901	43454	40345	32930	21006	2480
DGAW1.3	37021	36317	35330	31359	24677	12837	1062
DGAW2.1	35286	34718	34219	31536	26683	13876	2649
DGAW2.2	48005	46887	45803	41840	32645	15377	2840
DGAW2.3	33737	33195	32438	29451	24144	14367	3052
DGAW3.1	43980	43563	43096	40645	34505	15701	1595
DGAW3.2	61191	60764	59649	57459	54535	50117	1000
DGAW3.3	46186	45108	44030	38885	31233	18193	1541

Table S2. Distribution of bacterial sequences among taxonomic levels in rhizsophere soil of four *Ferula* species. Abbreviations: HDAW, *F. syreitschikowii*; XJAW, *F. gracilis*; DSAW, *F. ferulaeoides*; and DGAW, *F. lehmannii*. The samples names were labeled using a two two-number system, where the first number indicates the depth (1, 2, and 3 represent the 3, 20, and 40 cm depths, respectively) and the second number represents the replicate number.

		Proteobacteria	Actinobacteria	Cyanobacteria	Gemmatimonadetes	Bacteroidetes	Acidobacteria	Firmicutes	Verrucomicrobia	Thaumarchaeota	Planctomycetes	Factor 1	Factor 2	Factor 3	Factor 4
Proteobacteria	Sig.		0	0.002	0.038	0.051	0.004	0.416	0.008	0.047	0	0.861	0.381	0.59	0.133
Actinobacteria	Sig.	0		0.003	0.103	0.372	0.867	0.476	0.563	0.004	0.004	0.953	0	0.363	0.616
Cyanobacteria	Sig.	0.002	0.003		0.119	0.005	0.019	0.483	0.043	0.122	0.009	0.891	0.848	0.445	0.327
Gemmatimonadetes	Sig.	0.038	0.103	0.119		0.004	0.001	0.804	0	0.495	0.155	0.441	0	0.467	0.314
Bacteroidetes	Sig.	0.051	0.372	0.005	0.004		0.022	0.514	0.011	0.332	0.155	0.852	0.121	0.045	0.242
Acidobacteria	Sig.	0.004	0.867	0.019	0.001	0.022		0.111	0	0.525	0.003	0.269	0.016	0.131	0.295
Firmicutes	Sig.	0.416	0.476	0.483	0.804	0.514	0.111		0.18	0.073	0.115	0.287	0.098	0.186	0.741
Verrucomicrobia	Sig.	0.008	0.563	0.043	0	0.011	0	0.18		0.383	0.001	0.826	0.047	0.295	0.178
Thaumarchaeota	Sig.	0.047	0.004	0.122	0.495	0.332	0.525	0.073	0.383		0.063	0.152	0.094	0.007	0.269
Planctomycetes	Sig.	0	0.004	0.009	0.155	0.155	0.003	0.115	0.001	0.063		0.575	0.612	0.37	0.309
Factor 1	Sig.	0.861	0.953	0.891	0.441	0.852	0.269	0.287	0.826	0.152	0.575		1	1	1
Factor 2	Sig.	0.381	0	0.848	0	0.121	0.016	0.098	0.047	0.094	0.612	1		1	1
Factor 3	Sig.	0.59	0.363	0.445	0.467	0.045	0.131	0.186	0.295	0.007	0.37	1	1		1
Factor 4	Sig.	0.133	0.616	0.327	0.314	0.242	0.295	0.741	0.178	0.269	0.309	1	1	1	

Table S3. Correlation analysis of 4 principal components of 12 physicochemical factors with the relative abundance of bacteria in the rhizosphere (phylum level).

		Gammaproteobacteria	Chloroplast	unidentified_Gemmatimonadetes	Alphaproteobacteria	Thermoleophilia	unidentified_Acidobacteria	unidentified_Actinobacteria	Acidimicrobiia	Cytophagia	Sphingobacteriia	Factor 1	Factor 2	Factor 3	Factor 4
Gammaproteobacteria	Sig.		0.001	0.003	0.009	0.001	0.004	0.043	0.024	0.062	0.01	0.817	0.831	0.615	0.045
Chloroplast	Sig.	0.001		0.102	0.181	0.01	0.054	0.095	0.025	0.023	0.053	0.875	0.82	0.444	0.328
unidentified_Gemmatimonadetes	Sig.	0.003	0.102		0.439	0.37	0	0.153	0.12	0.091	0	0.441	0	0.467	0.314
Alphaproteobacteria	Sig.	0.009	0.181	0.439		0.872	0.447	0.118	0.237	0.219	0.462	0.692	0.149	0.55	0.079
Thermoleophilia	Sig.	0.001	0.01	0.37	0.872		0.793	0.002	0.074	0.198	0.692	0.98	0.014	0.093	0.789
unidentified_Acidobacteria	Sig.	0.004	0.054	0	0.447	0.793		0.194	0.092	0.971	0	0.3	0.005	0.143	0.68
unidentified_Actinobacteria	Sig.	0.043	0.095	0.153	0.118	0.002	0.194		0.015	0.641	0.058	0.75	0.003	0.583	0.456
Acidimicrobiia	Sig.	0.024	0.025	0.12	0.237	0.074	0.092	0.015		0.001	0.117	0.821	0.011	0.01	0.157
Cytophagia	Sig.	0.062	0.023	0.091	0.219	0.198	0.971	0.641	0.001		0.282	0.843	0.599	0.001	0.277
Sphingobacteriia	Sig.	0.01	0.053	0	0.462	0.692	0	0.058	0.117	0.282		0.453	0.053	0.261	0.268
Factor 1	Sig.	0.817	0.875	0.441	0.692	0.98	0.3	0.75	0.821	0.843	0.453		1	1	1
Factor 2	Sig.	0.831	0.82	0	0.149	0.014	0.005	0.003	0.011	0.599	0.053	1		1	1
Factor 3	Sig.	0.615	0.444	0.467	0.55	0.093	0.143	0.583	0.01	0.001	0.261	1	1		1
Factor 4	Sig.	0.045	0.328	0.314	0.079	0.789	0.68	0.456	0.157	0.277	0.268	1	1	1	

Table S4. Correlation analysis of 4 principal components of 12 physicochemical factors with the relative abundance of bacteria in the rhizosphere (class level).
